# Mental health benefits of a 1-week intensive multimodal group program for adolescents with multiple adverse childhood experiences

**DOI:** 10.1016/j.chiabu.2021.105349

**Published:** 2021-10-07

**Authors:** Susana Roque-Lopez, Elkin Llanez-Anaya, María Jesús Álvarez-López, Megan Everts, Daniel Fernández, Richard J. Davidson, Perla Kaliman

**Affiliations:** a Association Innocence In Danger Colombia (IIDC), 33 Avenue Saint Charles, 34090 Montpellier, France; b Universidad de Santander, Facultad de Ciencias Médicas y Salud, Instituto de Investigación Masira, Bucaramanga, Colombia; c Jacinto Benavente 3, 08100 Mollet del Valles, Spain; d Serra Húnter fellow. Department of Statistics and Operations Research & IMTech. Universitat Politècnica de Catalunya ⋅ BarcelonaTech (UPC). CIBERSAM. Spain; e Center for Healthy Minds, University of Wisconsin-Madison, 625 W. Washington Ave., Madison, WI 53703, United States of America; f Universitat Oberta de Catalonia (UOC), Rambla del Poblenou, 156, 08018 Barcelona, Spain

**Keywords:** Adverse childhood experience, Adolescence, PTSD, Integrative, Mindfulness, EMDR

## Abstract

**Background:**

Adverse childhood experiences (ACEs) are associated with a wide range of diseases, unsafe behavior and shorter life expectancy. However, there is scarce evidence on effective interventions for children or adolescents who report multiple ACEs, including abuse, neglect and household dysfunction.

**Objective:**

The aim of this study was to evaluate the mental health outcomes of a multimodal program designed for adolescents with multiple ACEs.

**Participants:**

Forty-four girls (aged 13–16 years, mean ACE score > 5) were randomized to an intervention group or a care-as-usual control group.

**Methods:**

The intervention included mindfulness-based practices, expressive arts and EMDR (Eye Movement Desensitization and Reprocessing Integrative) group treatment. We used questionnaires for adolescents to assess trauma (SPRINT, CPSS) and attention/awareness-related outcomes (MAAS-A) at baseline (T1), post-intervention (T2) and two-months post-discharge (T3).

**Results:**

Linear mixed effects model analyses showed significant Group by Time interactions on all the scales (F = 11.0, *p* = 0.015; F = 12.5 *p* < 0.001; and F = 6.4, *p* = 0.001, for SPRINT, CPSS and MAAS-A, respectively). After completing the program, the intervention group showed significant reduction in trauma-related outcomes (SPRINT, Δ%_(T2-T1)_ = −73%, *p* < 0.001; CPSS, Δ%_(T2-T1)_ = −26%, *p* < 0.001) while attention/awareness-related outcomes were improved by 57% (p < 0.001). These changes remained stable two months after discharge. SPRINT and CPSS scales were highly correlated (*r* = 0.833, *p* < 0.001) and outcomes from both trauma-related scales negatively correlated with mindfulness scores (MAAS-A/SPRINT, *r* = −0.515, *p* = 0.007; MAAS-A/CPSS, *r* = −0.553, *p* < 0.001).

**Conclusions:**

Results presented here support this multimodal group intervention as a feasible and promising program for reducing the psychological burden in adolescents with a history of multiple ACEs.

## 1. Introduction

Adverse childhood experiences (ACEs) are potentially traumatic events that occur during childhood or adolescence. ACEs include experiencing violence, abuse, or neglect, witnessing violence in the home or community, growing up in a household with substance use problems, mental health problems or instability due to parental separation or incarceration. Globally, up to 1 billion children aged 2–17 years experience physical, sexual, or emotional violence or neglect (Hillis et al., 2016). ACEs increase the risk of morbidity and mortality and can have a negative impact on life opportunities and social behavior throughout the life course. Exposure to ACEs also predicts an acceleration in cell aging as revealed by shortened telomeres (Li et al., 2017) and faster-running epigenetic clocks (Tang et al., 2020). Importantly, some of the strongest associated effects of multiple ACEs (e.g. violent behavior and addiction) increase the risk of ACEs across generations through intergenerational transmission of maltreatment and neglect (Widom et al., 2015).

Reporting 4 or more ACEs, constitutes a major risk factor for many health conditions, including overweight or obesity, diabetes, cancer, heart disease, respiratory disease, mental ill health, smoking, sexual risk taking, problematic alcohol and drug use and interpersonal and self-directed violence (Hughes et al., 2017). Therefore, efforts to improve and standardize tools and methods for young people who have experienced multiple victimization are a priority to reduce the suffering and health risks associated with ACEs (Oral et al., 2016). However, evidence-based interventions to improve outcomes in children and adolescents with multiple ACEs are still scarce (Campbell, 2020). Recent studies show encouraging clinical outcomes in child victims of trauma with multimodal programs that combine several therapies such as cognitive behavioral therapy, cardiovascular exercise, yoga, music, art, EMDR, individual counselling, and interactions with animals (Silverstone et al., 2016). In addition to the potential cumulative benefits of combining different approaches, it may be possible that multimodal programs allow children and adolescents to benefit from the therapists and methods that are more suitable for them, which is not an option with single therapeutic interventions.

Here, we explored the impact of a multimodal group program for adolescents with a history of multiple ACEs in which we combined three main components shown to improve mental health and behavior in youth, i.e. trauma-sensitive mindfulness-based practices (Ortiz & Sibinga, 2017), artistic expression (Malchiodi, 2005) and the EMDR (Eye Movement Desensitization and Reprocessing) group treatment protocol (Jarero & Artigas, 2012). We intentionally combined these three complementary strategies in a precise sequence with the aim of increasing the capacity to promote an overall effect. The program starts with mindfulness training and creative arts, activities that have been described to enhance attentional and emotional regulation, reduce anxiety and increase the capacity of expression and adaptation to the environment in youth. Recent randomized-controlled trials have shown that mindfulness-based programs for children and adolescents in schools or healthcare settings significantly increased resilience, lowered depressive symptoms and improved socio-emotional functioning (Dunning et al., 2019; Volanen et al., 2020); decreased anxiety symptoms and increased emotion regulation (Cotton et al., 2020) and decreased social anxiety and suicidal ideation (Lu et al., 2019). Moreover, in individuals with a childhood maltreatment history, a mindfulness-based intervention improved non-attachment and empathy, which contributed to reduce interpersonal distress and rejection sensitivity (Joss et al., 2020). The components of mindfulness training that may promote trauma recovery mainly include attentional regulation, focus on the “present moment”, and equanimity (a non-judgemental state toward experiences, whether they are pleasant, unpleasant, or neutral) (Desbordes et al., 2014). In addition to mindfulness training, the intervention presented here included creative and playful activities, which are useful tools to help young people express their emotional and cognitive universe while discovering the possibility to adapt their narratives in healthier directions (Malchiodi, 2005; Stewart et al., 2016). The third therapeutic ingredient of this multimodal program is EMDR, a method strongly recommended for PTSD treatment by the International Society for Traumatic Stress Studies guidelines for the treatment of children and adolescents with post-traumatic stress symptoms (Forbes et al., 2020). The standard EMDR protocol consists in focusing on selected sensations, emotions or memories while responding to bilateral visual, tactile, or auditory sensory stimulation (Shapiro, 1989). Although the neural mechanisms responsible for EMDR’s beneficial effects are poorly understood, this therapy seems to reconsolidate painful memories in less salient ways, allowing their adaptive resolution (Calancie et al., 2018; Gunter & Bodner, 2008). The EMDR group protocol approach (also known as EMDR Integrative Group Treatment Protocol, the Group Butterfly Hug Protocol or the Children’s EMDR Group Protocol) is an adaptation of the original EMDR protocol that has been proven effective in large groups of survivors of natural disasters, helping adults and young people with a range of psychological difficulties, including trauma, anxiety and depression (Jarero & Artigas, 2012).

Here we describe the protocol and mental health outcomes of this multimodal program in a group of 44 at-risk girls from 13 to 16 years old, placed in residential or semi-residential childcare settings due to inadequate parental care. Participants were randomized to an intervention group or a care-as-usual waiting list control group. We hypothesized that if effective, this program would decrease trauma-related outcomes in adolescents with multiple ACEs.

## 2. Methods

### 2.1. Participants

**Fig. 1.**
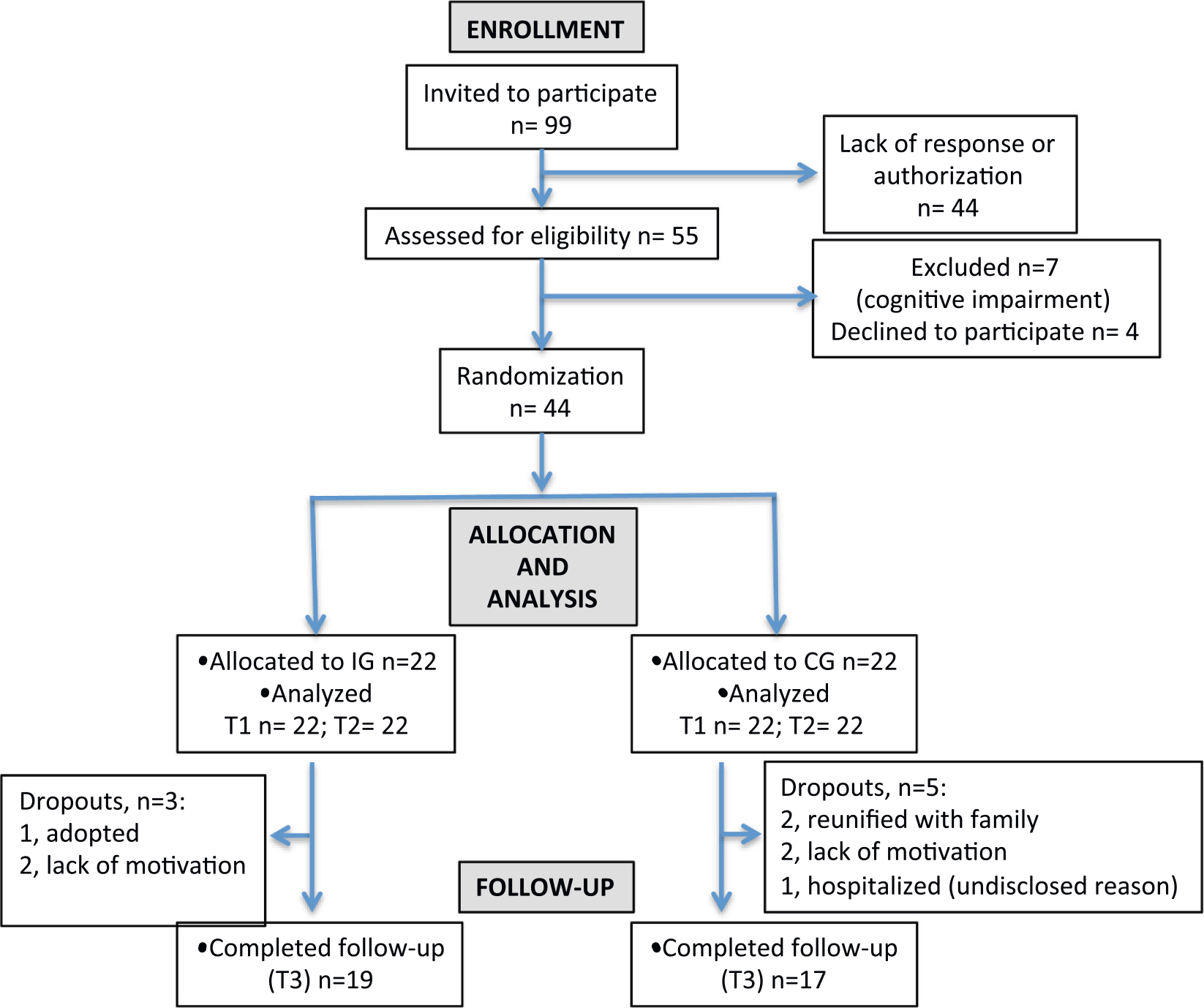
Participant flow diagram (CG: control group; IG: intervention group).

Recruitment was performed through the Colombian Institute of Family Well-Being (ICBF) in Bucaramanga (Colombia), where all interviews were conducted. All participants were females, aged 13–16 years, protected by ICBF-dependent residential or semi-residential youth care settings due to inadequate parental care, such as abuse and neglect. Exclusion criteria were life-threatening suicidality or self-harming behavior within the last 6 months, cognitive impairment, any documented pervasive developmental or psychiatric disorder or current substance dependence. Adolescents who met the inclusion criteria were invited for further baseline assessment. Fig. 1 shows the flowchart of participants invited, screened, enrolled and completing the study. A total of 44 subjects (*n* = 22/group) completed baseline (T1) and post-intervention (T2) tests. Follow-up assessment (T3, 2-months post-intervention) were completed by 36 subjects (*n* = 17 in the control group; *n* = 19 in the intervention group). Dropout reasons are described in Fig. 1. Table 1 provides the participants’ demographic information that we could collect. Due to the local regulations, we had no access to comprehensive information about family, health or life history of the participants from their legal representatives.

When the intervention was conducted, four subjects from each group were receiving medication (Control group: 1- fluoxetine; 2- sertraline; 3- lithium; 4- methylphenidate, sertraline and trazodone. Intervention group: 1- risperidone, valproic acid; 2- trazodone; 3- fluoxetine, trazodone; 4- risperidone, methylphenidate, valproic acid). These subjects continued with their treatments during our study. The participants had no previous experience with cognitive-behavioral, mindfulness-based or EMDR approaches.

Participants were randomized into two groups through a random-number generator, and underwent parallel and identical assessments at baseline, post-intervention and 2 months after the end of the intervention. To participate in this research, participants and their legal representatives provided a written informed consent.

All participants received full information about the intervention when they signed the informed consent. For ethical reasons and to prevent feelings of rejection or frustration in the subjects assigned to the control group, the informed consent indicated that the control group was invited to attend the same program at the end of the study. Participants were informed to which group they had been assigned after the basal assessment (T1). All the assessments were performed at the respective residential or non-residential youth care centers from which participants were recruited.

### 2.2. Intervention

A summary of the program’s schedule on a day-by-day basis is described in the Supplementary information (Table S1a). On day 1, activities were focused on creative and playful activities to facilitate the integration of the participants. From day 2 to 8, there was an early morning routine starting with an awakening with soft music and a hot beverage in the garden. This was followed by a 30 min session of initiation to Ashtanga Yoga, an aerobic practice aimed to tone and strengthen the muscles, calm the mind and increase concentration. This practice has been shown to provide physical and mental health benefits in children and adolescents (Benavides & Caballero, 2009; Culver et al., 2015). The yoga practice ended with a short relaxation and a guided meditation to cultivate positive affective states (i.e. loving kindness and compassion), based on Thich Nhat Hanh teachings (Nhat Hanh, 2012). The early morning session was followed by a healthy breakfast, after which the adolescents attended a mindfulness practice based on Eline Snel’s “Mindfulness Matters” program (Michel et al., 2019; Snel, 2012), an adaptation for children and adolescents of the mindfulness-based stress reduction (MBSR) curriculum (Kabat-Zinn, 2013). Mindfulness practices consisted in simple exercises to help teenagers stay present and develop the qualities of curiosity and benevolence in relation to their own physical and emotional feelings, including mind-body exercises to reduce stress and increase resilience (e.g. slow deep breathing with an expiration that is long relative to the inspiration (Eichhammer et al., 2012)); guided imagery such as ‘The Inner safe place’, ‘The inner child’ and ‘The tree’ (Zehetmair et al., 2019) and ‘The butterfly hug’ or ‘Hug of self love’, a self-administered bilateral stimulation (BLS) method for self-soothing (Artigas & Jarero, 2005). Daily activities included artistic expression through art and craft, music, dance and dramatic play. These activities were based on themes such as reawaken a sense of wonder, healing wounds as a way to freedom and the benefits of engaging in a healthy and compassionate way toward oneself and others.

On days 5 and 6, the adolescents attended two EMDR group protocol sessions/day to reprocess traumatic memories. After completion of the group sessions, if required, individual EMDR therapy was provided. The day before the first EMDR group protocol session (day 4), participants attended a puppet show based on the story “The Extraordinary Momentum for Discovery and Reconciliation of Buddy, the brown dog that everyone called grumble” (Meignant & Meignant, 2007). This story was used to explain the internal turmoil after a trauma and to introduce the EMDR therapy through the story of a puppy-dog victim of maltreatment.

To promote a joyful and relaxed environment and build cohesion in the group, the program also included two movie-night sessions featuring films about adolescence, resilience, love and friendship. Following the same rationale, there were 2 special dinners, a “Welcome party”, on day 2 and a “Celebration party” on day 7.

The intervention was performed during a school holiday week (20–27 June 2019) and it was conducted at a nature retreat facility in Santander, Colombia. During that same week, the control group was engaged in holiday activities proposed by youth care centers, all of them regulated by the ICBF. Table S1b describes the schedule of these holiday activities provided by ICBF. While some activities were similar between groups (e.g., physical exercise, dance, acting, games, movies), the control group schedule did not include any approach to specifically address traumatic experiences or attentional and emotional regulation.

All the assessments reported in the study were performed between July and October 2019. All analyses reported are based on participants who started and completed the intervention.

### 2.3. Therapeutic team

A psychologist and a psychiatrist, both of them certified in EMDR therapy and trained in mindfulness-based interventions for children and adolescents, led the program. An expressive arts therapist coordinated the artistic expression activities. All of the instructors had a vast experience in working with children and adolescents with ACEs. During the same week, the control group’s activities were guided by a school teacher, an art and craft teacher, a psychologist, a social worker and an occupational therapist.

### 2.4. Measures

#### 2.4.1. Adverse childhood experience (ACE)

We used the standard ACE questionnaire to assess for a history of ten adverse exposures: (1) emotional abuse, (2) physical abuse, (3) sexual abuse, (4) emotional neglect, (5) physical neglect, (6) separation from biological parents (7) witnessing domestic violence, (8) household substance abuse, (9) mental illness in household, and (10) having incarcerated family members. Questions were adapted to Spanish from the Behavioral Risk Factor Surveillance System ACE module and Violence Against Children Surveys (Behavioral Risk Factor Surveillance System Survey ACE Data, n.d.; Chiang et al., 2016). Response options were dichotomous (1 = yes, 0 = no). Total score ranged from 0 to 10, higher scores indicated exposure to more ACEs.

#### 2.4.2. Short PTSD Rating Interview (SPRINT)

The SPRINT scale comprises 8 items, including questions addressing PTSD’s core symptoms (Connor & Davidson, 2001). Items 1 to 4 assess criteria of intrusive re-experiencing, avoidance, numbing and hyperactivity. Items 5 and 6 refer to depression and stress tolerance. Items 7 and 8 refer to performance in daily activities and social functioning. This scale was translated from English to Spanish and from Spanish to English, revised and validated (Jarero et al., 2013).

In this scale, participants rate how often each symptom has occurred in the last week and each item is rated on a 5-point scale: 0 (not at all), 1 (a little), 2 (moderately), 3 (quite), and 4 (very). Scores between 18 and 32 correspond to marked or severe symptoms of PTSD, scores 11–17 correspond to moderate symptoms, scores 7–10 are for mild symptoms, and scores of 6 or less indicate no or minimal PTSD symptoms. The SPRINT performs similarly to the Clinician-Administered PTSD Scale (CAPS) in assessing PTSD symptom clusters and can be used as a diagnostic tool (Vaishnavi et al., 2006).

#### 2.4.3. Child PTSD Symptom Scale (CPSS)

We used the validated Spanish version of the CPSS (Serrano-Ibáñez et al., 2018), one of the most frequently used scales to assess PTSD in children and adolescents (Foa et al., 2001). The CPSS is composed by 17 items designed for children aged 8–18 years. Participants rate how often each symptom has occurred in the past month and each item is rated on a 4-point scale that ranges from 0 (not at all) to 3 (5 or more times a week). The total score is the sum of all items. It has been described that dysphoria four-factor model fit well in children and adolescents using the CPSS in both Spanish and English (items 1–5: intrusion; items 6–8: avoidance; items 9–15: dysphoria; items 16–17: arousal) (Meyer et al., 2015).

#### 2.4.4. Mindful Attention Awareness Scale-Adolescents (MAAS-A)

We used the Spanish version (Calvete et al., 2014) of the MAAS for adolescents (MAAS-A) (Brown & Ryan, 2003) that consists of 14 items with responses on a 6-point scale (1 = almost always, 2 = very frequently, 3 = somewhat frequently, 4 = somewhat infrequently, 5 = very infrequently, 6 = almost never), assessing attention/awareness-related experiences (being aware or not of feelings, sensations, thoughts or behaviors). Higher scores reflect higher mindful attention and awareness trait.

### 2.5. Data analysis

All the analyses were conducted in the statistical software R (R Core Team, 2020). Demographics differences between groups were evaluated by Mann-Whitney U or Student’s *t*-test. Demographic differences in age, body mass index (BMI), number of reported ACEs (ACEs) and amount of time spent in youth care centers (Months in youth care center) between the intervention group (IG) and the control group (CG) were evaluated by Mann-Whitney U or Student’s *t*-test. Categorical differences between groups in the youth care centers from which they were recruited (Center of origin) were evaluated by Pearson’s chi-squared test (Table 1). A linear regression model for binary outcomes (ACE questionnaire) was performed for pairwise comparisons, in which the log odds of the outcomes were modeled as a linear combination of the predictor variables. The reported odds ratios were computed by raising Euler’s constant (e) to the coefficients resulted from the logistic regression. We used a mixed effects model to evaluate the intervention effects on the psychometric scales using the lme4 package in R (Bates et al., 2015). Test scores were used as the response variable and group and time points were the independent variables. Subject random effects were established in order to minimize inter-subject unknown baseline differences. Group × Time interactions were the effect of interest.

Participant ID was established into the mixed model as a random effect to take into account the variability of the population, to fix possible bias due to the structure of the sample, and to prevent spurious associations derived from the analysis (3–4SI Fig. S1). In addition, our mixed effect model assumes that the individual specific effects are uncorrelated with the independent variables. Fixed effects were group allocation (intervention vs. control) and time as a longitudinal variable (SI Table S4). No influence of demographic covariates (age, body mass index, number of ACEs and fostering institution) in the outcome variable was detected. Tukey’s post-hoc comparisons were conducted to analyse changes across time within each group using age, body mass index, number of ACEs and fostering institution as covariates. Partial correlation analyses to evaluate the relationships between scales were performed using data from all participants in both groups after controlling for group and time. For the correlation analyses, missing data were excluded in a pairwise way. Multinomial logistic regression was used to determine the odds ratios (OR) between types of ACEs. Cronbach’s alpha coefficients (Cronbach, 1951) were computed to assess the internal consistency of the scales (MAAS-A, CPSS and SPRINT) (SI Table S5, A and B). Variance-covariance matrices for MAAS-A, SPRINT and CPSS scales are shown in SI Table S6.

## 3. Results

### 3.1. Frequency and patterns of ACEs

**Fig. 2.**
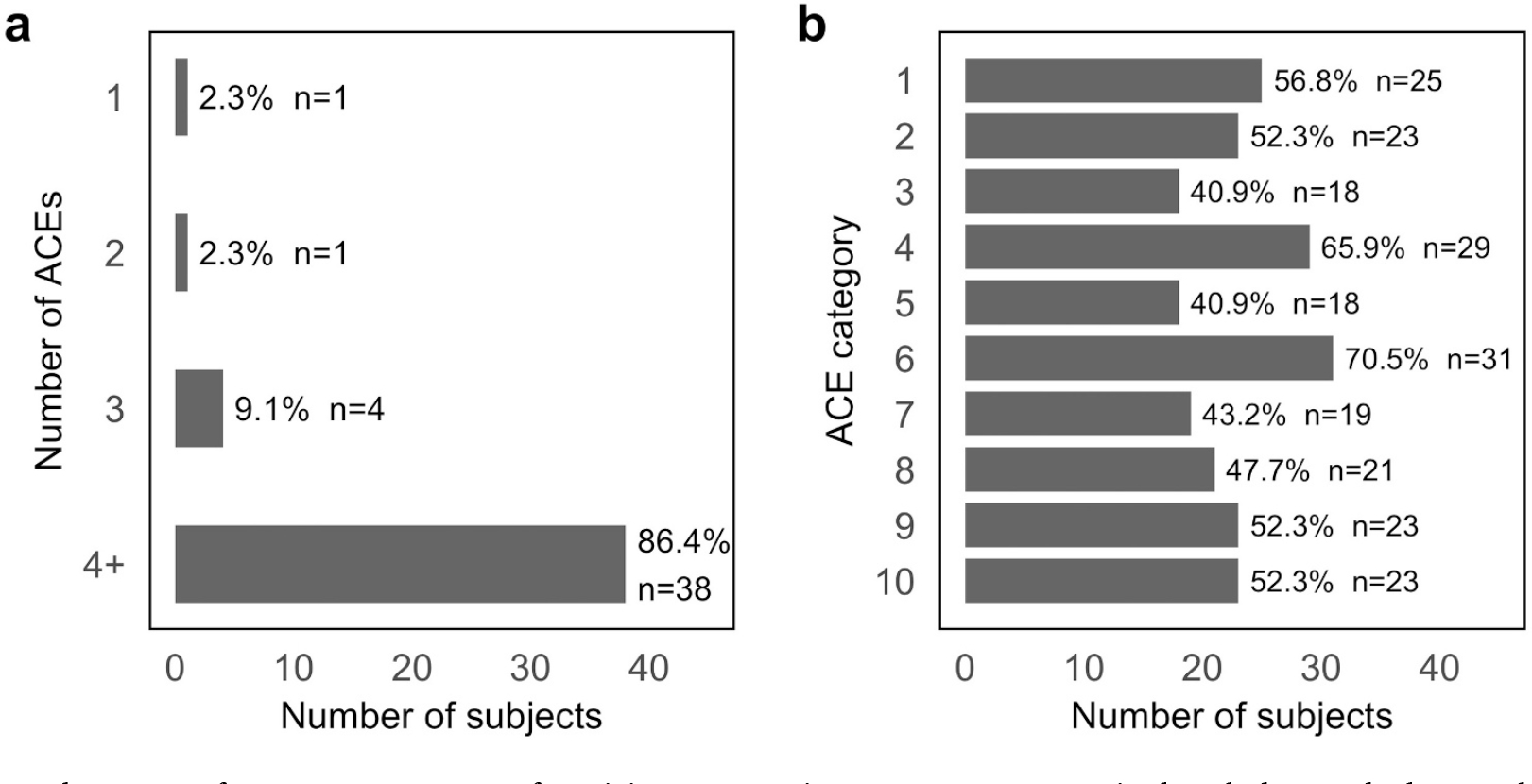
Frequency and patterns of ACEs. a. Percentage of participants reporting 1, 2, 3 or ≥4 ACEs in the whole sample. b. Prevalence rate for each ACE category in the whole sample, with the percentage of positive responses by item. ACE categories are: (1) emotional abuse, (2) physical abuse, (3) sexual abuse, (4) emotional neglect, (5) physical neglect, (6) separation from biological parents (7) witnessing domestic violence, (8) household substance abuse, (9) mental illness in household, and (10) having incarcerated family members.

All participants reported at least one ACE, with 86.4% reporting 4 or more types of ACEs. The mean number of ACE was 5.1 in the control group (CG) and 5.4 in the intervention group (IG) (Fig. 2a). Fig. 2b indicates the prevalence rate for each ACE category, with the percentage of positive responses by item in the total sample. Biological parent separation and emotional neglect were the most prevalent ACEs (70.5% and 65.9%, respectively). Pairwise associations between types of ACEs showed two strong positive associations (SI Table S2). In subjects who were exposed to drug abuse in the household, the odds of being exposed to domestic violence was 11.9 times higher than in subjects who were not exposed to substance abuse in the household (*p* < 0.001). In subjects who were emotionally abused, the odds of being physically abused were 7.2 times higher than in subjects who were not emotionally abused (*p* < 0.01). On the other hand, physical abuse and biological parent separation show slight but significant negative association (OR 0.2; *p* < 0.05), suggesting that the presence of one reduces the odds of the other event.

### 3.2. Short PTSD Rating Interview (SPRINT)

Table 2 shows the mean total scores for the intervention (IG) and control (CG) groups at baseline (T1), post-intervention (T2) and 2-months follow-up (T3), as well as a mixed effects model analysis, which indicates Group (intervention vs. waitlist control), Time (T1, T2 and T3) and Group × Time interactions. The mean total score at baseline (T1) was similar between groups (18.2 (9.7) in the CG and 18.1 (6.2) in the IG), both being above the PTSD threshold score of 14–17 proposed for this scale (Connor & Davidson, 2001). While in the IG, mean scores at T2 and T3 dropped to 4.8 (6.0) and 4.4 (4.8), respectively, in the control group these values remained above the PTSD threshold score at both T2 and T3 (14.3 (9.4) and 16.6 (8.7), respectively). A mixed effects model analysis revealed significant effects in Group, Time and Group × Time interactions (F = 11.0, *p* = 0.002; F = 40.0, *p* < 0.001; F = 16.1, *p* < 0.001, respectively). The internal consistency of this scale was good (T1) and strong (T2 and T3), as measured by Cronbach’s alpha coefficients (SI, Table S4a).

**Fig. 3.**
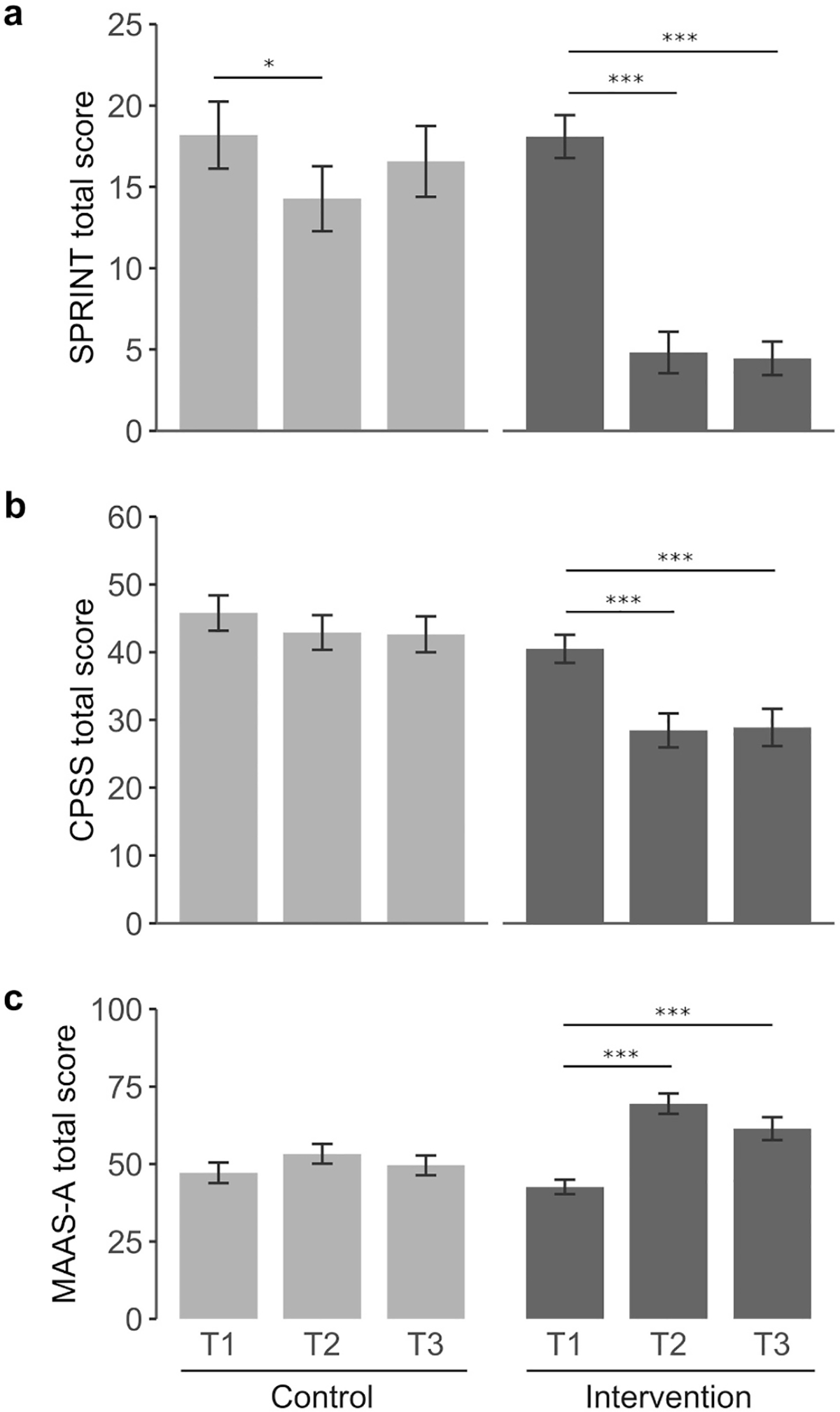
Changes in self-report outcome measures in the intervention and control group. Tukey’s post-hoc comparisons were conducted to analyse changes across time in all scales. (a) SPRINT, (b) CPSS and (c) MAAS-A. Light grey (control group); dark grey (intervention group). Mean scores are indicated for each scale. Baseline (T1), post-intervention (T2) and 2-months follow-up (T3). *p < 0.05; ***p < 0.001.

Post-hoc analyses revealed a significant decrease in SPRINT total score in response to the intervention both at T2 and T3 (IG: T2-T1, −13.3 (1.5) *p* < 0,001; T3-T1, −13.7 (1.5) *p* < 0.001). While remaining above the PTSD threshold score of 14–17, the CG showed a significant reduction in SPRINT total score at T2, which was no maintained at T3 (CG: T2-T1, −3,9 (1.5) *p* = 0.031; T3-T1, −1.6 (1.7) *p* = 0.578) (Fig. 3a; SI Table S3).

### 3.3. Child PTSD Symptom Scale (CPSS)

**Fig. 4.**
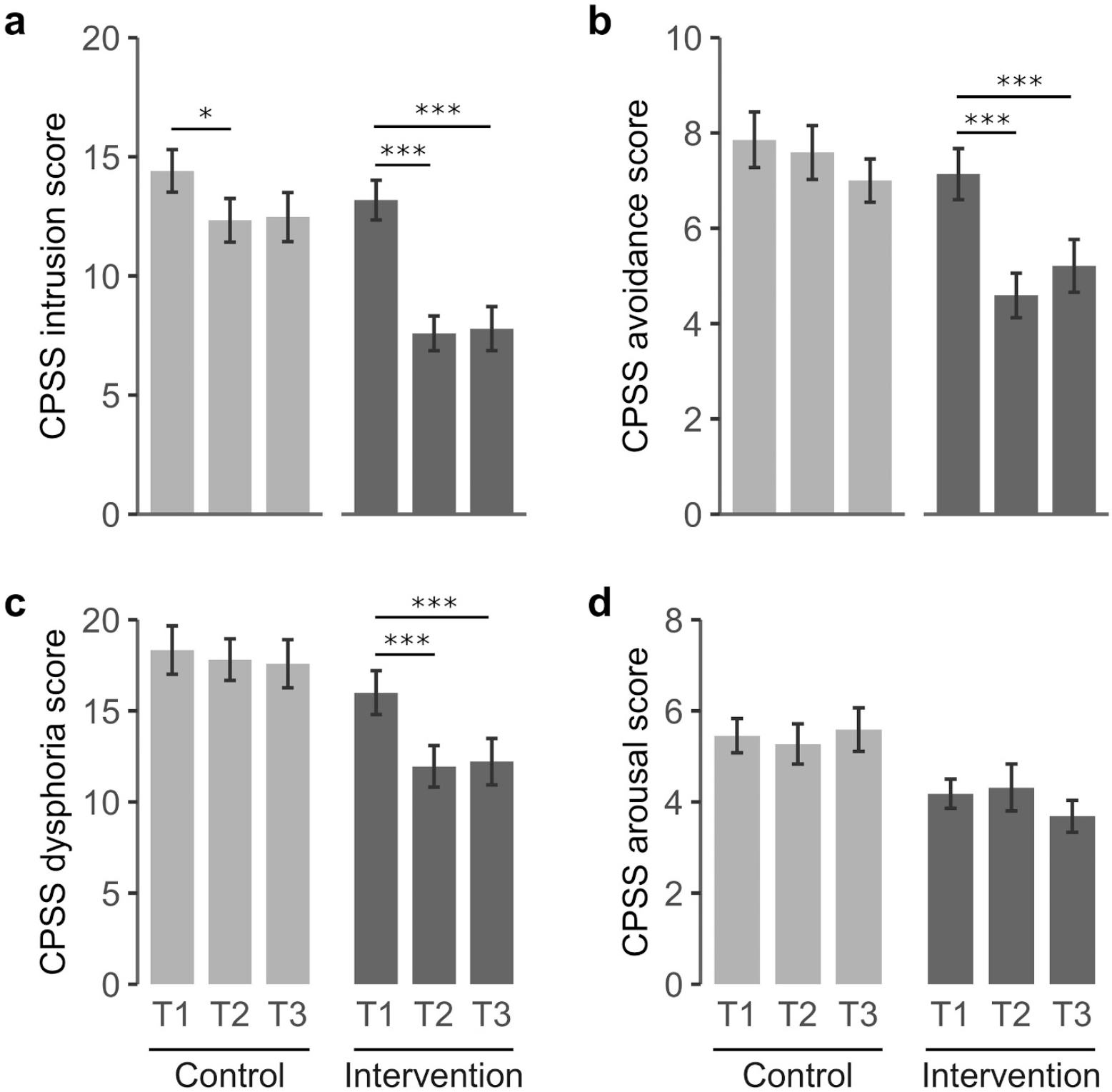
Changes in the four-factor dysphoria model of the CPSS. Tukey’s post-hoc comparisons were conducted to analyse changes across time in intrusion (a), avoidance (b), dysphoria (c), and arousal (d). Light grey (control group); dark grey (intervention group). Mean scores are indicated for each scale. Baseline (T1), post-intervention (T2) and 2-months follow-up (T3). *p < 0.05; ***p < 0.001.

CPSS total score showed significant Group, Time and Group × Time interactions (F = 12.5 *p* = 0.001; F = 13.2 *p* < 0.001; and F = 4.7 *p* = 0.012, respectively), with significant effects for intrusion, avoidance and dysphoria dimensions in all analyses (Table 2). Arousal scores showed a significant Group interaction (F = 9.6 *p* = 0.004) but the effects on Time and Group × Time were not significant. In the IG, CPSS post-hoc analyses showed a significant decrease in total score post-intervention that was maintained at T3 (IG: T2-T1, −12.0 (2.3) *p* < 0.001; T3-T1, −11.6 (2.4) *p* < 0.001) (Fig. 3b; SI Table S3). Fig. 4 shows the changes in the dysphoria four-factor model across time for each group. The intrusion dimension showed a significant reduction at T2 in the control group but this effect was no longer significant in the follow-up (T2-T1, −2.1 (0.8) *p* = 0.039; T3-T1, −1.9 (0.8) *p* = 0.077). In the IG, the reduction on the intrusion score was more pronounced than in the CG and it was maintained at T3 (T2-T1: −5.6 (0.8) *p* < 0.001; T3-T1: −5.4 (0.8) *p* < 0.001). Avoidance and dysphoria were significantly decreased in the IG at T2 and T3 while no change was detected for these dimensions in the control group across time. The arousal dimension remained unchanged in both groups. The CPSS total scores and the intrusion and dysphoria subscales presented acceptable internal consistency according to Cronbach’s alpha measures. In contrast, the arousal and avoidance subscales showed poor internal consistency at T1 and T3, probably due to the small number of test items (SI, Table S4a and b).

### 3.4. Mindful Attention Awareness Scale-Adolescents (MAAS-A)

The mixed effects model analysis showed significant Group, Time, and Group × Time interaction effects (F = 6.4, *p* = 0.015; F = 20.0 *p* < 0.001; and F = 7.7, *p* = 0.001, respectively) on the MAAS-A scale (Table 2). The internal consistency of this scale was poor at T1 but good/strong at T2 and T3, as measured by Cronbach’s alpha coefficients (SI, Table S4a).

Tukey post-hoc comparisons revealed a significant increase in total score post-intervention in the IG that was maintained at 2-month follow-up (IG: T2-T1, 26.9 (3.7) *p* < 0.001; T3-T1, 18.8 (3.9) *p* < 0.001). No significant change across time was detected in the CG (Fig. 3c; SI Table S3).

### 3.5. Correlation analyses

Cross correlation analyses showed that mindfulness-related scores negatively correlated with PTDS-related outcomes (MAAS-A/SPRINT, *r* = −0.515, *p* = 0.007; MAAS-A/CPSS, *r* = −0.553, *p* < 0.001) and that both PTSD-related scales (SPRINT and CPSS) were highly correlated with each other (*r* = 0.833, *p* < 0.001) (Table 3).

## 4. Discussion

There is a growing body of evidence linking ACE exposure to poor physical and mental health throughout the lifespan. ACE screening in primary care has been proposed as a preventive strategy, considering that a regular contact between clinicians, families and children can build trusting relationships that may help identify and treat the toxic consequences of ACEs (Gilgoff et al., 2020). Early ACE screening can be useful to identify asymptomatic youth and may help stop abuse and mitigate the development of physical and mental diseases (Gordon et al., 2020; Harris, 2020). However, it has been argued that a major barrier for the successful implementation and outcomes of ACE screening in primary care is the scarce evidence on interventions for children or adolescents who report multiple victimization (Campbell, 2020). Here we describe an intensive multimodal group program, with encouraging outcomes in a randomized controlled intervention with adolescent girls reporting a mean ACE score > 5. The program presented here highly decreased PTSD-related symptoms and enhanced attention/awareness-related outcomes. In the intervention group, the beneficial effects of the program (T2-T1) were maintained over a 2-month follow-up period (T3-T1). These data support the outcomes from our previous uncontrolled pilot studies using this multimodal program in children and adolescents with a history of sexual and physical abuse (Jarero et al., 2013).

In this study, 86.4% of participants reported 4 or more categories of ACEs, a level of trauma exposure which is found with a prevalence between 2 and 15% in the general population (Hughes et al., 2017; Kessler et al., 2010; Merrick et al., 2018). Previous studies show that multimodal methods of treatment provide significant long-term benefits across different mental health domains in children and adolescents exposed to adverse experiences (Silverstone et al., 2016). Here, we intentionally combined different therapeutic strategies as a means to better address the diversity of factors associated with multiple ACEs. The main goal of our program was to decrease the distress and emotional burden of painful memories and to strengthen internal resources, trust and resilience. To this end, the intervention included several sessions per day of mindfulness-based practices and expressive arts activities. In the program’s schedule, these components preceded by several days the EMDR group protocol sessions in order to enhance the participant’s attentional and emotional regulation and their adaptation to the environment before addressing the reprocessing of traumatic experiences.

We observed a significant increase in mindfulness total score after the intervention which represents an encouraging finding considering that MAAS-A scores have been negatively correlated with symptoms of depression, anger, antisocial behavior, substance dependence and lack of self-regulation (Calvete et al., 2014). Supporting these findings, we observed a negative correlation between MAAS-A score and PTSD-related outcomes, which were assessed using a short questionnaires (SPRINT) and a more compelling scale (CPSS) for adolescents. We found a high positive correlation between the mean scores of these two PTSD scales (*r* = 0.833, *p* < 0.001). The main advantage of the 8-item SPRINT scale is that it takes on average 5–10 min to complete with no appreciable loss in psychometric strength. However, the CPSS scale was more informative than the SPRINT scale as it retrieved information on the four-factor dimensions of PTSD described in the DSM-V (intrusion, avoidance, dysphoria, arousal). However, due to very low internal consistency of the arousal and avoidance subscales, only the intrusion and dysphoria subscales were reliable in our study (SI Table S4b).

In conclusion, our results support this intervention as a promising integrative/complementary short-term program for reducing the psychological burden in adolescents with a history of multiple ACEs. Although we found improved psychological functioning across an extended post-discharge period of 2 months, the adolescents may still need group or individual follow-up support in order to enhance and strengthen the mental health benefits from this intervention. Future prospective studies to assess whether this program can contribute to reduce or prevent the negative health effects of multiple ACEs are warranted.

## 5. Limitations

A limitation of our study is the small sample size. Due to the location of our study and the characteristics of the sample, we could only recruit 44 participants (22/group). To minimize possible baseline differences between groups that could confound the outcome of the intervention, we randomized the participants into two groups that underwent parallel and identical assessments at baseline, post-intervention and 2 months after the end of the intervention. In addition, our intervention included a homogeneous group of subjects that may have helped to increase our capacity to detect an overall effect of the intervention, although the limitation of a convenience sample is a low generalizability of the results. Finally, a post-hoc power analysis (ClinCalc calculator), using the CPSS total scores (Table 2), indicated that the power of the study was 98,2% at T2 and 96% at T3 (alpha risk = 0.05, beta risk = 0.20).

Immediately after discharge, the participants from the intervention group participants returned to their respective youth care institutions where they met the control group subjects. Therefore, there was contact between groups before the post-intervention assessment (T2, 1 week after the intervention) and the follow-up assessment (T3, 2 months after the intervention). In the context of our study, this was an unavoidable limitation as participants from both groups shared youth care centers. However, the intervention’s effects in T2 remained stable 2 months later (T3), suggesting a poor influence of contact between groups on the study outcomes.

The intervention described here is comprehensive in terms of ingredients. The current study did not intend to disentangle the effect of each particular ingredient of this multimodal program. To this end, future studies will require the design of active control groups that may help to eventually simplify this promising but complex intervention.

## Supplementary Material

Supplementary Data

## Figures and Tables

**Table 1:** Participant’s demographic data. Body mass index (BMI), number of reported ACEs (ACEs), amount of time spent in youth care centers (Months in care center), youth care centers from which participants were recruited (Center of origin), control group (CG) and intervention group (IG).

	CG					IG					Statistic test	
	*n*	Mean	SD	Min	Max	*n*	Mean	SD	Min	Max	Statistic	*p*-Value

Age	22	14.1	1.2	12.0	16.0	22	14.0	1.1	12.0	16.0	244.50	0.96
BMI	22	22.3	2.5	16.5	27.4	22	21.4	2.5	18.5	28.3	1.14	0.26
ACEs	22	5.1	1.6	1.0	8.0	22	5.4	1.8	3	9.0	−0.52	0.60
Months in care center	15	30.9	49.9	1	175	18	26.8	32.0	2	116	114.50	0.64
Center of origin	22	–	–	–	–	22	–	–	–	–	3.76	0.84

**Table 2:** Mean total scores in self-report measures in the intervention group and control group. Mean total score values for the intervention (IG) and control groups (CG) at baseline (T1), post-intervention (T2) and 2-months follow-up (T3) are indicated. Mixed effects model analysis indicates Group (intervention vs. control), Time (T1, T2 and T3) and Group × Time interactions.

Scale	CG			IG			Mixed model					
	T1	T2	T3	T1	T2	T3	Group		Time		Group × Time	
	Mean (SD)	Mean (SD)	Mean (SD)	Mean (SD)	Mean (SD)	Mean (SD)	*F*	*p*-Value	*F*	*p*-Value	*F*	*p*-Value

SPRINT	18.2 (9.7)	14.3 (9.4)	16.6 (8.7)	18.1 (6.2)	4.8 (6.0)	4.4 (4.8)	11.0	0.002	40.0	<0.001	16.1	<0.001
CPSS	46.1 (12.2)	43.0 (12.0)	42.7 (10.1)	40.5 (9.7)	28.5 (11.7)	28.9 (12.0)	12.5	0.001	13.2	<0.001	4.7	0.012
Intrusion	14.4 (4.2)	12.3 (4.2)	12.5 (4.2)	13.2 (3.9)	7.6 (3.4)	7.8 (4.0)	9.6	0.003	28.7	<0.001	6.3	0.003
Avoidance	7.9 (2.7)	7.6 (2.6)	7.0 (1.9)	7.1 (2.5)	4.6 (2.2)	5.2 (2.4)	11.8	0.001	6.2	0.003	3.1	0.051
Dysphoria	18.3 (6.1)	17.8 (5.2)	17.6 (5.4)	16 (5.6)	12.0 (5.3)	12.2 (5.5)	9.2	0.004	5.6	0.006	4.4	0.015
Arousal	5.5 (1.8)	5.3 (2.1)	5.6 (2.0)	4.2 (1.5)	4.3 (2.4)	3.7 (1.5)	9.6	0.004	0.2	0.848	0.6	0.569
MAAS-A	47.2 (14.3)	53.3 (14.6)	49.6 (13.2)	42.6 (10.4)	69.5 (15.4)	61.4 (16.1)	6.4	0.015	20.0	<0.001	7.7	0.001

**Table 3:** Cross correlation among scores of all self-report measures. Partial correlation analyses to evaluate the relationships between scales were performed using data from all participants in both groups after controlling for group and time. Mindfulness-related outcomes (measured by the MAAS-A scale) negatively correlated with PTSD-related outcomes (measured by SPRINT and CPSS scales). Both PTSD-related scales (SPRINT and CPSS) were highly correlated with each other.

	MAAS-A		SPRINT		CPSS	
	*r*	*p*-Value	*r*	*p*-Value	*r*	*p*-Value

MAAS-A	–	–	−0.515	0.007	−0.553	<0.001
SPRINT	−0.515	0.007	–	–	0.833	<0.001
CPSS	−0.553	<0.001	0.833	<0.001	–	–
