# Supplementary material for: Mental health benefits of a 1-week intensive multimodal group program for adolescents with multiple adverse childhood experiences
Source: Child Abuse Negl. Author manuscript; Available in PMC 2021 Dec 1. (PMC8627589; doi:10.1016/j.chiabu.2021.105349)
Supplement: Supplementary Data [file NIHMS1750289-supplement-Supplementary_Data.pdf]

Supplementary information

Mental health benefits of a 1-week intensive multimodal group program for adolescents with multiple adverse childhood experiences.

Table S1a. 1-week intensive multimodal program for multiple ACE schedule.

Time	Day 1	Day 2	Day 3	Day 4	Day 5	Day 6	Day 7	Day 8
**6:00 - 6:45 am**		Awakening with soft music, warm shower and hot beverage						
**6.45-7.30 am**		Initiation to yoga + short guided meditation						
**7:30 - 8:30 am**		Healthy breakfast						
**8:30 - 9:00 am**		Mindfulness for adolescents.						
**9:00 - 11:45 am**	Arrival	Mind-body techniques (includes a break for a healthy snack and beverage)			EMDR Group Protocol Session 1	EMDR Group Protocol Session 3	Integrative arts (acting, dance, music) (if required: EMDR- IGTP in small group and / or EMDR individuald)	Farewell & Departure
**11:45 am - 12:30 pm**	Welcoming and presentation of activities.	Swimming pool						
**12:30 pm - 1:30 pm**	Healthy lunch							
**1:30 - 2:30 pm**	Free Time							
**2.30 - 4.30 pm**	Integrative arts (acting, dance, music, plastic arts)				EMDR Group Protocol Session 2	EMDR Group Protocol Session 4	Artistic performance rehearsal	
**4.30-5.30 pm**	Free time/healthy snack and beverage							
**5.30-6.30 pm**	Integrative arts (acting, dance, music)	Dialogues: healthy food, lifestyle, experiences, sexuality, internet, etc				Group mandala painting	Celebration party: mandala exhibit; scenic performance by participants; closing ceremony	
**6:30 -7:00 pm**	Mind-body techniques							
**7.00 pm**	Healthy dinner	Wellcome party	Healthy dinner	Healthy dinner and puppet show IEP Colombia	Healthy dinner- inspirational movie night	Healthy dinner		
**between 9-11 pm**	Bed time							

Table S1b. Control group daily activities (school holiday season).

Time	Activities
6-8 am	wake up, room cleaning, shower and breakfast
8-10 am	physical exercise
10-11 am	holiday homework
11-12 am	acting, dance, plastic arts
12 am - 12.30 pm	table games
12.30-2 pm	lunch and free time
2-4pm	manual activity, e.g. needlework
4-5 pm	cinema forum, reading club
5-6 pm	physical exercise
6-7 pm	dinner
7-7.30 pm	spiritual activity, e.g. prayer or bible reading
8 pm	sleep time

Table S2. Odds ratio and 95% confidence interval for pairwise associations between types of ACEs

	1. Emotional abuse	2. Physical abuse	3. Sexual abuse	4. Emotional neglect	5. Physical neglect	6. Parent separation	7. Domestic violence	8. Substance abuse	9. Mental illness	10. Incarcerated member
	OR (95% CI)	OR (95% CI)	OR (95% CI)	OR (95% CI)	OR (95% CI)	OR (95% CI)	OR (95% CI)	OR (95% CI)	OR (95% CI)	OR (95% CI)
1. Emotional abuse		7.2 (2.0; 30.1)**	0.6 (0.2; 2.1)	1.2 (0.3; 4.4)	1.3 (0.4; 4.7)	0.3 (0.1; 1.1)	1.1(0.3; 3.7)	1.0 (0.3; 3.4)	1.4 (0.4; 4.8)	1.0 (0.3; 3.2)
2. Physical abuse	7.2 (2.0; 30.1)**a		0.6 (0.2; 2.0)	1.4 (0.4; 5.0)	1.3 (0.4; 4.3)	0.2 (0.0; 0.9)	1.5 (0.4; 5.1)	2.1 (0.6; 7.3)	1.4 (0.4; 4.8)	1.4 (0.4; 4.8)
3. Sexual abuse	0.6 (0.2; 2.1)	0.6 (0.2; 2.0)		1.1 (0.3; 3.9)	0.9 (0.2; 3.0)	0.5 (0.1; 1.8)	2.4 (0.7; 8.4)	1.7 (0.5; 5.9)	0.6 (0.2; 2.0)	1.3 (0.4; 4.3)
4. Emotional neglect	1.2 (0.3; 4.4)	1.4 (0.4; 5.0)	1.1 (0.3; 3.9)		1.6 (0.5; 6.3)	0.5 (0.1; 1.9)	0.4 (0.1; 1.2)	0.5 (0.1; 1.7)	2.1 (0.6; 7.9)	0.6 (0.2; 2.2)
5. Physical neglect	1.3 (0.4; 4.7)	1.3 (0.4; 4.3)	0.9 (0.2; 3.0)	1.6 (0.5; 6.3)		1.2 (0.3; 4.6)	2.4 (0.7; 8.4)	2.5 (0.7; 9.0)	0.9 (0.3; 2.9)	0.6 (0.2; 2.0)
6. Biological parent separation	0.3 (0.1; 1.1)	0.2 (0.0; 0.9)*c	0.5 (0.1; 1.8)	0.5 (0.1; 1.9)	1.2 (0.3; 4.6)		0.3 (0.1; 1.3)	0.5 (0.1; 1.7)	0.6 (0.1; 2.2)	0.9 (0.2; 3.4)
7. Domestic violence	1.1(0.3; 3.7)	1.5 (0.4; 5.1)	2.4 (0.7; 8.4)	0.4 (0.1; 1.2)	2.4 (0.7; 8.4)	0.3 (0.1; 1.3)		11.9 (3.1; 56.3)***	1.0 (0.3; 3.4)	2.2 (0.7; 7.7)
8. Substance abuse	1.0 (0.3; 3.4)	2.1 (0.6; 7.3)	1.7 (0.5; 5.9)	0.5 (0.1; 1.7)	2.5 (0.7; 9.0)	0.5 (0.1; 1.7)	11.9 (3.1; 56.3)***b		0.7 (0.2; 2.3)	3.1 (0.9; 11.2)
9. Mental illness	1.4 (0.4; 4.8)	1.4 (0.4; 4.8)	0.6 (0.2; 2.0)	2.1 (0.6; 7.9)	0.9 (0.3; 2.9)	0.6 (0.1; 2.2)	1.0 (0.3; 3.4)	0.7 (0.2; 2.3)		1.0 (0.3; 3.3)
10. Incarcerated member	1.0 (0.3; 3.2)	1.4 (0.4; 4.8)	1.3 (0.4; 4.3)	0.6 (0.2; 2.2)	0.6 (0.2; 2.0)	0.9 (0.2; 3.4)	2.2 (0.7; 7.7)	3.1 (0.9; 11.2)	1.0 (0.3; 3.3)	

* p value <0.05; ** p value <0.01; *** p value <0.001

Table S3. Tukey's post-hoc comparisons were conducted to analyse changes across time within each group using age, body mass index and number of ACEs and fostering institution as covariates. Baseline (T1), post-intevention (T2) and 2-months follow-up (T3) are indicated.

	**Control group** Change, (SE) *p-value*			**Intervention group** Change, (SE) *p-value*		
**Scale**	T2-T1	T3-T1	T3-T2	T2-T1	T3-T1	T3-T2
**SPRINT**	*-3.9, (1.5)* *0.031*	*-1.6, (1.7)* *0.578*	*2.3, (1.7)* *0.398*	*-13.3, (1.5)* *<0.001*	*-13.7, (1.5)* *<0.001*	*-0.4, (1.5)* *0.969*
**CPSS**	*-2.9, (2.3)* *0.420*	*-3.4, (2.5)* *0.435*	*-0.3, (2.5)* *0.996*	*-12.0, (2.3)* *<0.001*	*-11.6, (2.4)* *<0.001*	*0.4, (2.4)* *0.871*
intrusion	*-2.1, (0.8)* *0.039*	*-1.9, (0.8)* *0.077*	*0.2, (0.8)* *0.994*	*-5.6, (0.8)* *<0.001*	*-5.4, (0.8)* *<0.001*	*0.2, (0.8)* *0.718*
avoidance	*-0.3, (0.7)* *0.898*	*-0.9, (0.7)* *0.359*	*-0.6, (0.7)* *0.591*	*-2.5, (0.6)* *<0.001*	*-1.9, (0.7)* *0.020*	*0.6, (0.7)* *0.557*
dysphoria	*-0.5, (1.0)* *0.991*	*-0.7, (1.1)* *0.992*	*-0.2, (1.1)* *1.000*	*-4.0, (1.0)* *<0.001*	*-3.8, (1.0)* *0.002*	*0.2, (1.0)* *0.949*
arousal	*-0.2, (0.5)* *0.930*	*0.1, (0.5)* *0.994*	*0.3, (0.5)* *0.900*	*0.1, (0.5)* *0.960*	*-0.5, (0.5)* *0.692*	*-0.6, (0.5)* *0.529*
**MAAS-A**	*6.1, (3.8)* *0.229*	*2.4, (4.0)* *0.725*	*-3.7, (4.0)* *0.696*	*26.9, (3.7)* *<0.001*	*18.8, (3.9)* *<0.001*	*-8.1, (3.8)* *0.106*

Table S4. Fixed effect estimators of mixed model for MAAS-A, SPRINT and CPSS scales.
Estimators, standard deviation, 95% confidence interval and p-value of the fixed effects for mixed models are shown. There is a significant effect of group x time interaction for the three scales studied; the positive estimator indicates an increase in the slope in MAAS-A scale and a decrease in the slope in SPRINT and CPSS scales. Group and time effects separately are not significant with the exception of T2 in SPRINT scale.

	**Fixed effects**			
	Estimator	SD	[95% CI]	*p-value*
**MAAS-A**				
Intercept	46.96	3.27	[40.66, 53.25]	<0.001
Group (Intervention)	-3.91	4.54	[-12.64, 4.86]	0.390
Time – T2	6.32	3.81	[-1.06, 13.67]	0.102
Time – T3	3.01	4.04	[-4.75, 10.88]	0.446
Group (Intervention) : T2	20.13	5.32	[9.87, 30.45]	<0.001
Group (Intervention) : T3	15.57	5.61	[4.76, 26.45]	<0.01
**SPRINT**				
Intercept	18.01	1.67	[14.78, 21.23]	<0.001
Group (Intervention)	0.09	2.34	[-4.44, 4.62]	0.971
Time – T2	-3.91	1.51	[-6.83, -0.98]	0.012
Time – T3	-1.70	1.69	[-4.98, 1.58]	0.319
Group (Intervention) : T2	-9.36	2.14	[-13.50, -5.23]	<0.001
Group (Intervention) : T3	-11.94	2.27	[-16.33, -7.55]	<0.001
**CPSS total**				
Intercept	45.69	2.50	[40.84, 50.55]	<0.001
Group (Intervention)	-5.19	3.52	[-12.01, 1.62]	0.144
Time – T2	-2.86	2.27	[-7.25, 1.52]	0.210
Time – T3	-3.08	2.48	[-7.88, 1.72]	0.219
Group (Intervention) : T2	-9.18	3.20	[-15.38, -2.98]	0.005
Group (Intervention) : T3	-7.77	3.44	[-14.44, -1.13]	0.026

Table S5. Cronbach alpha was computed to assess the internal consistency of scales.
a. Cronbach's alpha coefficients for MAAS-A, SPRINT and CPSS total scores.
b. Cronbach's alpha coefficients for CPSS subscale scores.
Cronbach's alpha coefficients that are less than 0.5 are usually unacceptable while very high values (i.e., > 0.95), may indicate redundancy in the scale items.

a

	Internal Consistency	
	Cronbach's α	[95% CI]
**MAAS-A**		
Time 1	0.56	[0.38, 0.82]
Time 2	0.91	[0.86, 0.94]
Time 3	0.85	[0.71, 0.91]
**SPRINT**		
Time 1	0.84	[0.70, 0.90]
Time 2	0.93	[0.88, 0.96]
Time 3	0.90	[0.83, 0.94]
**CPSS total**		
Time 1	0.87	[0.77, 0.91]
Time 2	0.94	[0.90, 0.96]
Time 3	0.93	[0.89, 0.95]

b

	Internal Consistency	
	Cronbach's α	[95% CI]
**CPSS intrusion**		
Time 1	0.78	[0.64, 0.87]
Time 2	0.87	[0.77, 0.92]
Time 3	0.90	[0.84, 0.94]
**CPSS avoidance**		
Time 1	0.49	[0.14, 0.69]
Time 2	0.74	[0.58, 0.84]
Time 3	0.46	[-0.07, 0.70]
**CPSS dysphoria**		
Time 1	0.83	[0.71, 0.90]
Time 2	0.86	[0.79, 0.91]
Time 3	0.87	[0.76, 0.92]
**CPSS arousal**		
Time 1	0.27	[-0.41, 0.63]
Time 2	0.78	[0.60, 0.89]
Time 3	0.53	[0.05, 0.77]

Figure S1. Random effect of random intercepts are shown for MAAS-A (a), SPRINT (b) and CPSS (c). 95% confidence intervals are depicted. Random effects show similar distribution and effect range; black points indicate confidence intervals that do not overlap with zero.

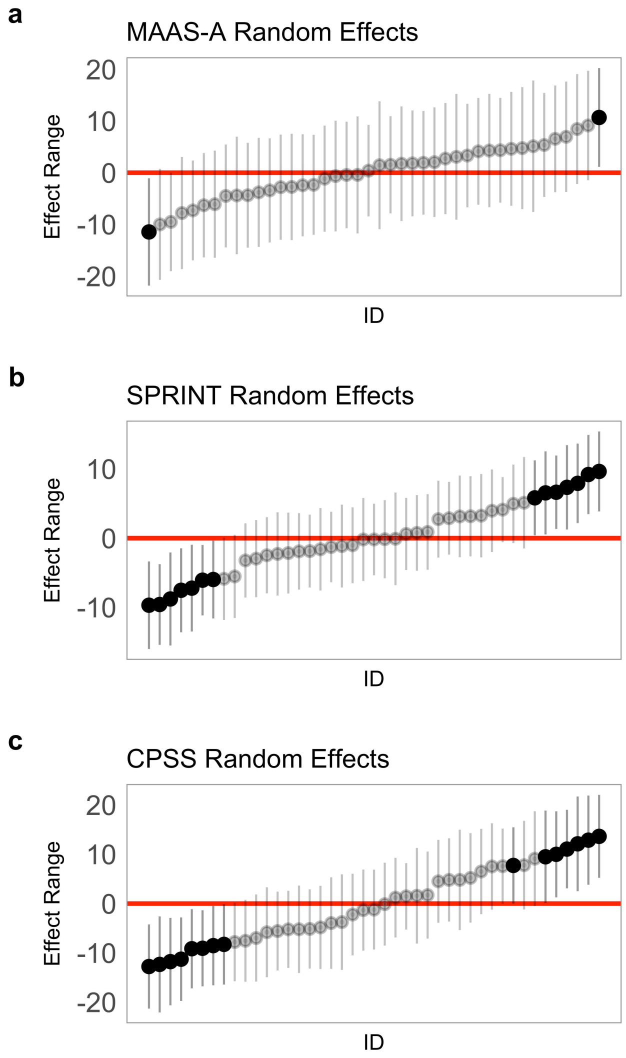

Table S6. Variance-covariance matrix for MAAS-A, SPRINT and CPSS scales.
Variance-covariance matrix is shown for all parameters (fixed, random effect, and residual) of the mixed models are depicted. Variances are indicated in the diagonal elements and covariances are located in the off-diagonal elements. The assumption of an unstructured variance-covariance matrix is applied to the 3 mixed models tested.

**MAAS-A**	Intercept	Group	Time T2	Time T3	Group x TimeT2	Group x TimeT3
Intercept	10.66	-10.66	-7.72	-7.62	7.72	7.62
Group	-10.66	20.58	7.72	7.62	-15.02	-14.94
Time T2	-7.72	7.72	14.52	7.63	-14.52	-7.63
Time T3	-7.62	7.62	7.63	16.34	-7.63	-16.34
Group x TimeT2	7.72	-15.02	-14.52	-7.63	28.32	14.95
Group x TimeT3	7.62	-14.94	-7.63	-16.34	14.95	31.45

**SPRINT**	Intercept	Group	Time T2	Time T3	Group x TimeT2	Group x TimeT3
Intercept	2.77	-2.77	-1.14	-1.13	1.14	1.13
Group	-2.77	5.46	1.14	1.13	-2.28	-2.28
Time T2	-1.14	1.14	2.28	1.14	-2.28	-1.14
Time T3	-1.13	1.13	1.14	2.87	-1.14	-2.87
Group x TimeT2	1.14	-2.28	-2.28	-1.14	4.57	2.28
Group x TimeT3	1.13	-2.28	-1.14	-2.87	2.28	5.15

**CPSS**	Intercept	Group	Time T2	Time T3	Group x TimeT2	Group x TimeT3
Intercept	6.27	-6.27	-2.57	-2.55	2.57	2.55
Group	-6.27	12.36	2.57	2.55	-5.13	-5.12
Time T2	-2.57	2.57	5.13	2.57	-5.13	-2.57
Time T3	-2.55	2.55	2.57	6.16	-2.57	-6.16
Group x TimeT2	2.57	-5.13	-5.13	-2.57	10.26	5.13
Group x TimeT3	2.55	-5.12	-2.57	-6.16	5.13	11.85
